# Practice of Offering a Small Pouch of Tobacco for Free With a Big Pouch of Pan Masala: A Strategic Move to Circumvent Gutkha Regulations

**DOI:** 10.34172/ijhpm.9537

**Published:** 2025-12-21

**Authors:** Vinay Kumar Gupta, Nishita Kankane, Seema Malhotra

**Affiliations:** ^1^Department of Public Health Dentistry, King George’s Medical University, Lucknow, India.; ^2^Community Health Centre, Lucknow, India.

## Dear Editor,

 Gutkha—a mixture of crushed areca nut, tobacco, catechu, paraffin wax, slaked lime, and flavoring agents—is a well-known smokeless tobacco product associated with a high risk of oral submucous fibrosis and oral cancer.^1-4^ Recognizing its carcinogenic potential, the Government of India banned the manufacture, sale, and distribution of Gutkha in 2012 under the Food Safety and Standards (Prohibition and Restrictions on Sales) Regulation, 2011 issued by the Food Safety and Standards Authority of India (FSSAI).^5^ This landmark decision aimed to protect public health and curb the alarming rise in oral cancer cases linked to smokeless tobacco use.

 However, a concerning trend has recently emerged wherein manufacturers offer a small pouch of tobacco “free” with a larger pouch of Pan Masala. This dual packaging or co-branding strategy effectively circumvents the Gutkha ban by exploiting a regulatory loophole: tobacco and Pan Masala are sold separately but intended to be used together, thereby recreating Gutkha at the point of consumption.^6^ This reflects the promotional intentions of tobacco companies to sustain consumption despite regulatory restrictions. Moreover, it exposes gaps in the implementation of specific tobacco control laws, enabling such practices to persist.

## Regulatory Circumvention

 By bundling tobacco with a non-tobacco product like Pan Masala, manufacturers bypass the explicit prohibition on “manufacture and sale of pre-mixed tobacco and Pan Masala” while continuing to ensure consumer access to the same addictive product. This twin-pack or combo-pack marketing tactic represents a deliberate circumvention of tobacco control laws and undermines the spirit of the Gutkha ban.^7^

## Public Health Implications

 This practice poses serious public health threats, including:

1. **Undermining regulatory effectiveness:** By exploiting legal loopholes, manufacturers dilute the impact of public health legislation.
2. **Reinforcing nicotine addiction:** Pan masala (a non-nicotine product) is widely advertised by celebrities and through sponsorship of major events, which increases its demand. At the point of sale, consumers often receive a combination of pan masala and tobacco products and fail to distinguish between what is advertised and what is actually marketed. This indirect pairing reinforces nicotine addiction.
3. **Promoting product normalization:** The co-marketing of Pan Masala and tobacco blurs boundaries between legal and banned products, sustaining social acceptability.
4. **Increased initiation risk:** The aggressive advertising of paan masala normalises such products for young people, who may purchase them assuming they are the same as the advertised brand. This confusion increases the likelihood of experimentation with tobacco-containing variants, ultimately raising the risk of becoming regular users.
5. **Scientific perspective:** From a scientific standpoint, this strategy represents a form of *marketing and product placement manipulation* used by the tobacco industry to bypass existing health regulations. It parallels tactics such as flavored product promotion, indirect advertising, and cross-brand incentives, all designed to maintain consumer interest and tobacco use while appearing to adhere to legal restrictions.

## The Way Forward

 To address this issue, the following measures are crucial:

1. **Strict enforcement** of the Gutkha ban, including penalties for surrogate sales or co-packaging.
2. **A definitive ban on surrogate advertising**, especially advertisements of Pan Masala that indirectly promote tobacco brands. This includes stricter monitoring of celebrity endorsements,^8^ event sponsorships, and brand extensions that mimic tobacco product identity. Strengthening these provisions is essential, as surrogate advertising continues to mislead consumers and normalize harmful products under the guise of legality.
3. **Clear enforcement of COTPA (Cigarettes and Other Tobacco Products Act, 2003)**^9^ **provisions—** particularly Section 5 (prohibition of direct and indirect advertising), which is frequently bypassed through surrogate promotion of pan masala that mirrors tobacco branding; and Section 7 (pictorial health warnings), which is diluted when products are co-packaged without appropriate warnings. Identifying regulatory gaps in monitoring surrogate advertising, cross-branding, and point-of-sale promotions represents an important area for future research.
4. **Amendments to COTPA and FSSAI regulations to explicitly prohibit:** (*a*) Cross-branding or brand sharing between tobacco and non-tobacco products, (*b*) Promotional bundling or complementary distribution, and (*c*) Same-brand celebrity endorsement across tobacco and non-tobacco categories.
5. **Public education campaigns** highlighting that offering free or complementary pouches is a marketing strategy aimed at sustaining use, particularly among price-sensitive consumers.
6. **Monitoring and surveillance** by State Food Safety Commissioners and enforcement agencies to detect such deceptive practices.

In conclusion, the practice of offering tobacco pouches free with Pan Masala undermines decades of tobacco control efforts in India. It highlights the need for regulatory vigilance, intersectoral coordination, and consumer awareness to prevent the tobacco industry from exploiting such loopholes. Sustained enforcement, coupled with behavioral interventions, is essential to protect current and future generations from the devastating health consequences of smokeless tobacco.

## Disclosure of artificial intelligence (AI) use

 ChatGPT has been used for outlining the content and checking grammer.

## Ethical issues

 Not applicable.

## Conflicts of interest

 Authors declare that they have no conflicts of interest.

## Disclaimers

 The views expressed in this letter to the editor are those of the authors and not of the institution or publisher.
